# Transcriptome analysis of divergent residual feed intake phenotypes in the *M. longissimus thoracis et lumborum* of Wannan Yellow rabbits

**DOI:** 10.3389/fgene.2023.1247048

**Published:** 2023-10-23

**Authors:** Dongwei Huang, Yuanlang Wang, Pingping Qi, Haisheng Ding, Huiling Zhao

**Affiliations:** Anhui Provincial Key Laboratory of Livestock and Poultry Product Safety Engineering, Institute of Animal Husbandry and Veterinary Medicine, Anhui Academy of Agricultural Sciences, Hefei, China

**Keywords:** feed efficiency, residual feed intake, rabbit production, transcriptome, differentially expressed gene, signaling pathway, *M. longissimus thoracis et lumborum*, meat quality

## Abstract

**Introduction:** Feed efficiency is an important economic trait in rabbit meat production. The identification of molecular mechanisms and candidate genes for feed efficiency may improve the economic and environmental benefits of the rabbit meat industry. As an alternative to the conventional feed conversion ratio, residual feed intake (RFI) can be used as an accurate indicator of feed efficiency.

**Methods:** RNA sequencing was used to identify the differentially expressed genes (DEGs) in the *M. longissimus thoracis et lumborum* of eight Wannan Yellow rabbits with excessively high or low RFIs (HRFI or LRFI, respectively). Thereafter, Gene Ontology (GO) analysis, enrichment using the Kyoto Encyclopedia of Genes and Genomes (KEGG) database, gene set enrichment analysis (GSEA), and protein–protein interaction (PPI) network analysis was conducted.

**Results:** In total, 445 DEGs were identified in the *M. longissimus thoracis et lumborum* of rabbits with high and low RFIs. The significantly enriched GO terms identified in these two groups were primarily involved in energy and mitochondrial metabolism and oxidation–reduction processes. KEGG analysis identified 11 significantly enriched pathways, including oxidative phosphorylation, PI3K-Akt signaling, and extracellular matrix-receptor interaction pathways. According to GSEA, the expressions of genes and pathways related to mitochondrial function were upregulated in HRFI rabbits, whereas genes with upregulated expressions in LRFI rabbits were related to immune response and energy metabolism. Additionally, PPI network analysis revealed five potential candidate genetic markers.

**Conclusion:** Comparative analysis of the *M. longissimus thoracis et lumborum* transcriptomes in HRFI and LRFI rabbits revealed *FOS*, *MYC*, *PRKACB*, *ITGA2*, and *FN1* as potential candidate genes that affect feed efficiency in rabbits. In addition, key signaling pathways involved in oxidative phosphorylation and PI3K-Akt and ECM-receptor interaction signaling impact rabbit feed efficiency. These findings will aid in breeding programs to improve feed efficiency and optimize RFI selection of rabbits for meat production.

## 1 Introduction

Rabbit meat is considered an excellent nutritional source owing to its high protein and low fat contents, high proportion of unsaturated fatty acids, and low cholesterol and sodium levels (Siddiqui et al., 2023). Feed cost accounts for 60% of total rabbit breeding costs and is therefore an important factor affecting overall rabbit production costs (Molette et al., 2016). Feed efficiency is one of the most economically and environmentally relevant aspects in rabbit meat production (Cesari et al., 2018). First proposed in 1963, residual feed intake (RFI) is defined as the difference between actual and predicted feed intake and expected feed requirements for the maintenance and growth of an animal over a specific period. RFI is generally considered the most suitable parameter for evaluating feed efficiency (Koch et al., 1963) and has been utilized for the artificial selection of feed efficiency in dairy cows (Connor et al., 2013), pigs (Barea et al., 2010), and poultry (Fathi et al., 2021). Previous research has identified RFI as a moderately inherited characteristic, which can improves feed efficiency in modern breeding (Sell-Kubiak et al., 2017). In rabbits, the genetic correlation between feed efficiency and growth rate is lower than that in other species. RFI can substantially increase rabbit growth rate within a few years (Blasco et al., 2018); therefore, identification of effective biomarkers to facilitate RFI selection is needed to shorten the selection process.

Feed efficiency is closely linked to energy metabolism (Patience et al., 2015; Fischer et al., 2018; Lancaster, 2021; Mota et al., 2022). Skeletal muscle is considered to be the main energy metabolic tissue in rabbits and plays an important role in regulating systemic homeostasis (Fan et al., 2021). Rabbit meat quality is dictated by muscle fiber type and characteristics that arise during skeletal muscle development (Du et al., 2022). The *M. longissimus thoracis et lumborum* is the largest erector spinae muscle and is commonly used for meat quality assessments in rabbits and other animals (Zotte et al., 2022), thus providing an ideal model to study feed efficiency.

Next-generation sequencing facilitates the screening of mechanisms underlying RFI to accelerate the breeding process. RNA sequencing (RNA-seq) is a widely applied and highly effective method used in livestock studies for comparing individuals with extreme trait phenotypes and identifying differentially expressed genes (DEGs) and pathways among groups of domesticated animals (Ramayo-Caldas et al., 2012; Ge et al., 2019; Xiao et al., 2021). However, most transcriptome studies on the molecular mechanisms underlying RFI differences have focused on cows (Salleh et al., 2017), pigs (Hou et al., 2020), and chickens (Xiao et al., 2021), whereas related studies on rabbits are scarce.

The Wannan Yellow rabbit, an indigenous Chinese breed native to the southern region of Anhui Province, China, is popular in the meat industry because of its high daily weight gain performance and feed efficiency in the early growth stage. Currently, indigenous rabbit farming for meat consumption is primarily conducted in rural areas. This provides economic opportunities for farmers and favorably impacts population maintenance in marginal areas (Siddiqui et al., 2023). Strategies to improve feed efficiency are essential for increasing the competitiveness of the rabbit breeding industry. In addition, the selection of feed-efficient rabbits helps maintain meat protein output while decreasing grain consumption and nutrient excretion to address the global food shortage. Thus, improving rabbit feed efficiency traits is an important strategy to increase the economic gain of farmers. Our three primary study objectives were: 1) Identify the divergence of the skeletal muscle transcriptomic profile in rabbits with extreme RFIs, 2) Elucidate the underlying biology of RFI by investigating key genes and pathways implicated in RFI divergence, and 3) Provide new insights into biomarkers for RFI selection in rabbits.

## 2 Materials and methods

### 2.1 Ethics statement

All animal experiments and study procedures were conducted in strict accordance with protocols approved by the Animal Care Advisory Committee of the Anhui Academy of Agricultural Sciences (AAAS 2022-17) and the “Guidelines for Experimental Animals” of the Ministry of Science and Technology (Beijing, China).

### 2.2 Rabbits and RFI calculation

All Wannan Yellow rabbits were bred at the Anhui Academy of Agricultural Sciences Experimental Farm, Jixi, China according to the standard breeding program. A total of 110 rabbits (same male and female) with similar body weight (BW) of approximately 500 g were selected and transferred to three-layered individual metal cages (40 cm × 35 cm × 50 cm) at 35 days of age. The main experiment began 65–95 days after the 30-day pre-experiment (the dietary adaption periods). Rabbits were fed daily with a basal diet for growing rabbits (10.5 MJ metabolizable energy/kg diet, including crude protein 16%, crude fiber 18%, crude ash 12%, calcium 1%, phosphorus 0.4%, lysine 0.6%, and H_2_O 14%) (The Composition of experimental diets is shown in the Supplementary Table S1) formulated without antibiotics and water was provided *ad libitum*.

The feed intake (FI) and BW of rabbits were measured at 65–95 days of age. The feed conversion ratio (FCR) was calculated using FI and body weight gain (BWG). Metabolic body weight (MBW^0.75^), BWG, and average daily body weight gain (ADG) and average daily feed intake (ADFI) per individual were calculated according to rabbit BW at 65 and 95 days. The RFI value was used to measure the feed efficiency using Equation (4):

$$\text{RFI}=\text{ADFI }–\;\left(b_{0}+b_{1}\text{ ADG}+b_{2}\;{\text{MBW}}^{0.75}\right)$$

where *b*_*0*_, *b*_*1*_, and *b*_*2*_ represent the regression intercept, partial regression coefficient of ADFI on MBW^0.75^, and the partial regression coefficient of ADFI on ADG, respectively. The RFI values were calculated using the regression procedure in SAS (version 9.4, SAS Inst. Inc., Cary, NC). Outliers were excluded from the data. All experimental groups were ranked by RFI, with the eight most extreme samples from the high (*n* = 4) and low (*n* = 4) RFI female rabbits selected as the high residual feed intake (HRFI) and low residual feed intake (LRFI) groups for RNA extraction, respectively. Animal performance data was expressed as the least square means ± standard error of the mean. A Student’s t-test was used to analyze the difference in feed efficiency between the HRFI and LRFI groups. A *p-*value <0.05 was considered statistically significant.

### 2.3 RNA extraction and RNA-seq

Rabbits in the HRFI and LRFI groups were humanely euthanized. The *M. longissimus thoracis et lumborum* were immediately collected, frozen in liquid nitrogen, and stored at −80°C until RNA extraction. Total RNA was extracted using TRIzol™ reagent (Invitrogen, Carlsbad, CA, United States) according to the manufacturer’s instructions. RNA quality was assessed using an Agilent 2100 Bioanalyzer (Agilent Technologies, Palo Alto, CA, United States) and verified using RNase-free agarose gel electrophoresis. Verified total RNA was sent to Gene *Denovo* Biotechnology Co., Ltd. (Guangzhou, China) for cDNA library construction and sequenced on an Illumina HiSeq 2500 platform; 125 bp paired-end reads were generated. The acquired data were submitted to the Sequence Read Archive (National Institute of Health, Bethesda, MD, United States) under the accession number PRJNA978018.

### 2.4 RNA-seq data analysis

FastQC software (version 11.5; http://www.bioinformatics. Babraham. ac. uk/projects/fastqc) was used to re-evaluate raw sequence read quality before read alignment. Clean reads were obtained by discarding adaptors, poly N, or low-quality reads. An index of the reference genome was built and paired-end reads were mapped to the *Oryctolagus cuniculus* genome sequence (OryCun 2.0) in the Ensembl database (http://www.ensembl.org/). Transcripts were quantified in fragments per kilobase million (FPKM), which was used to indicate gene expression patterns. Cufflinks v2.2.1 (Ghosh and Chan, 2016) was used to calculate the expected number of FPKM for each gene.

### 2.5 Identification of DEGs and bioinformatics analysis

The expression values and DEGs were determined using DESeq2 software (Love et al., 2014). Genes with a |fold-change| ≥ 1.5 and a *p*-value <0.05 were assigned as differentially expressed. Gene Ontology (GO) annotation and Kyoto Encyclopedia of Genes and Genomes (KEGG) pathway analyses were performed using the Database for Annotation, Visualization, and Integrated Discovery (https://david.ncifcrf.gov/) for exploration of DEG functions. A corrected *p*-value of <0.05 was considered statistically significant.

### 2.6 Gene set enrichment analysis (GSEA)

All expressed genes in both groups were analyzed via GSEA software (http://software.broadinstitute.org/gsea/downloads.jsp), based on C5. CC, C5. BP, C5. MP, and C2. CP KEGG gene set collections (MSigDB v7.1, broad institute, Cambridge, MA, United States) (https://www.gsea-msigdb.org/gsea/msigdb/index.jsp). All expressed genes were ranked according to the fold-change (HRFI/LRFI) between the HRFI and LRFI groups. For each gene set, the enrichment score was calculated with a full ranking that reflected gene set distribution in the list and the normalized enrichment score (NES) was determined using the signal-to-noise normalization method (Ma et al., 2011). Gene sets with an absolute NES values of >1 and false discovery rates (FDR) ≤0.05 were considered significantly enriched.

### 2.7 Protein–protein interaction (PPI) analysis of DEGs

DEGs were submitted to the Search Tool for Retrieval of Interacting Genes (STRING) database (https://string-db.org/) to predict gene interaction relationships (Szklarczyk et al., 2021); confidence scores >0.7 were defined as significant. The DEG PPI networks were generated using the open-source software Cytoscape v3.7.2 (Shannon et al., 2003). The CytoHubba application in Cytoscape was used to screen hub genes.

### 2.8 RNA-seq validation

Eight genes were selected at random to quantify their expression levels using real-time quantitative polymerase chain reaction (RT-qPCR) in the HRFI (*n* = 4) and LRFI (*n* = 4) groups. Transcript expression pattern reliability obtained via RNA-seq was subsequently validated. Total RNA from the *M. longissimus thoracis et lumborum* of rabbits was extracted using TRIzol (Invitrogen, Carlsbad, CA, United States) and reverse-transcribed into cDNA using a PrimeScript™ RT-PCR Kit (Takara, Dalian, China) according to the manufacturer’s instructions. The eight primer pairs used in this study are listed in Supplementary Table S2. The synthesized cDNA was used as a template for RT-PCR using the CFX96 Touch™ Real-Time PCR Detection System (Bio-Rad, Hercules, CA, United States). The RT-PCR reaction was performed by heating at 95°C for 3 min, followed by 40 cycles at 95°C for 5 s and 60°C for 30 s. Quantitative variation and relative fold changes were calculated according to the 2^−ΔΔCT^ method normalized with *GAPDH* (Livak and Schmittgen, 2001). Significant differences were analyzed using Student’s t-test in the SAS software v9.0 (SAS Institute, Inc., Cary, NC, United States); statistical significance was set at a *p*-value <0.05. All analyses were performed in triplicate.

## 3 Results

### 3.1 Animal performance and feed efficiency

Differences in RFI, FCR, ADFI, MBW0.75, and ADG are shown in Table 1. The RFI and FCR of the LRFI group were significantly lower than those of the HRFI group (*p-*value <0.05). The ADFI and ADG of the LRFI group were significantly higher than those of the HRFI group (*p-*value <0.05). Moreover, there was no significant difference in MBW^0.75^ between the two groups (*p-*value >0.05).

**TABLE 1:** Characterization of performance and feed efficiency traits (Least square means and SEM).

	HRFI	LRFI	*p-*value
RFI, g/d	14.99 ± 1.52	−14.04 ± 1.24	<0.001
FCR, g:g	9.63 ± 0.52	7.72 ± 0.64	<0.001
ADFI, g/d	219.50 ± 2.98	234.77 ± 4.83	<0.05
MBW^0.75^, g	359.22 ± 11.14	391.79 ± 12.93	0.093
ADG, g/d	19.79 ± 0.93	33.24 ± 2.63	<0.001

RFI, residual feed intake; FCR, feed conversion ratio; ADFI, average daily feed intake over the assessed feeding period; MBW^0.75^.

Mean of metabolic body weight; ADG, average daily gain over the assessed feeding period.

### 3.2 Summary of RNA-seq data

The HRFI and LRFI groups had 41,233,136 to 46,620,708 and 43,761,150 to 50,540,362 raw reads, respectively (Table 2). Filtering resulted in the following clean reads: (HRFI: 41,059,578 to 46,420,846; LRFI: 43,572,142 to 50,540,362 (for each library, per group)). The general Q30 (Phred quality score >30 and error rate <0.1%) percentage of the clean data was >94%. The average number of clean reads for each library was mapped using the *O. cuniculus* (OryCun 2.0) genome assembly, which resulted in a mean mapping efficiency of 68.48%.

**TABLE 2:** Characteristics of the reads from eight rabbits with high and low RFI.

Sample id	Raw reads	Clean reads	Clean ratio (%)	Clean reads Q30 (%)	Total mapped ratio (%)
HRFI1	44,548,910	44,320,546	99.49	94.59	70.74
HRFI2	45,697,988	45,501,938	99.57	94.72	69.49
HRFI3	41,233,136	41,059,578	99.58	94.49	69.10
HRFI4	46,620,708	46,420,846	99.57	94.55	68.65
LRFI1	43,761,150	43,572,142	99.57	94.16	69.24
LRFI2	50,540,362	50,307,108	99.54	94.03	68.40
LRFI3	44,001,962	43,802,348	99.55	94.52	65.50
LRFI4	46,836,370	46,586,534	99.47	93.93	66.75

### 3.3 Identification of DEGs

**FIGURE 1:**
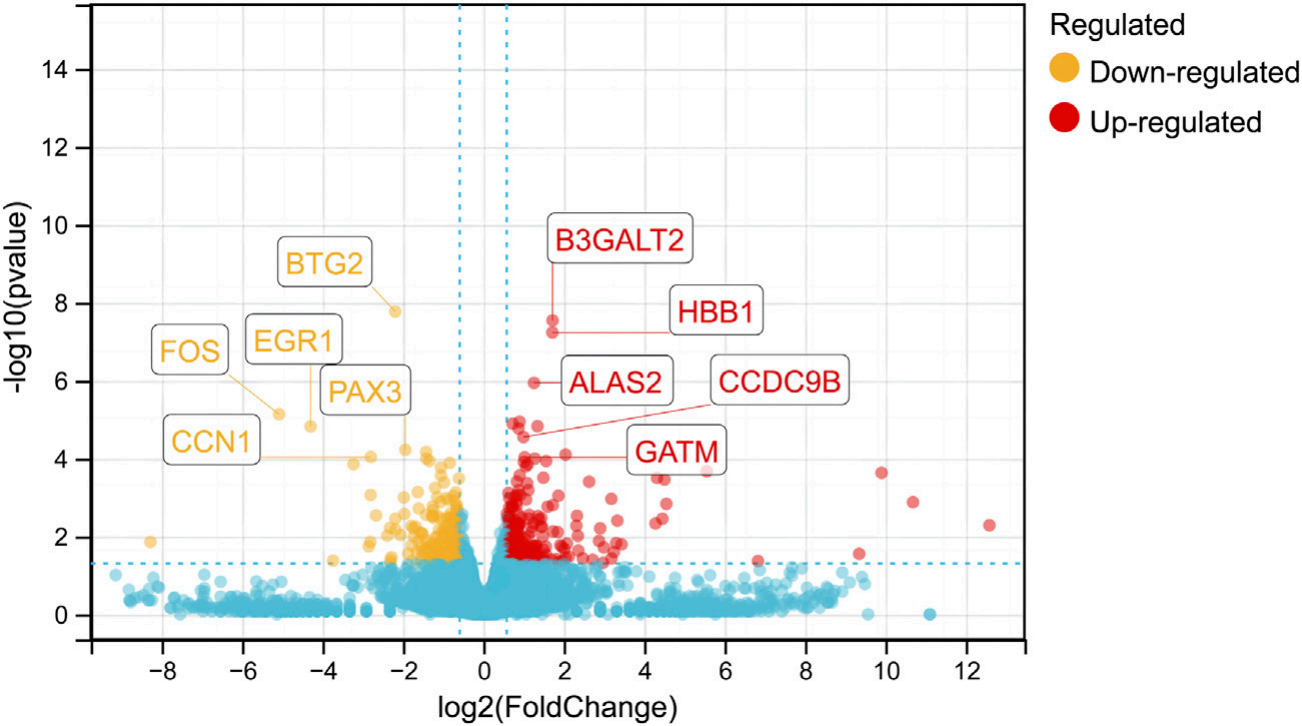
Analysis of differentially expressed genes (DEGs) between the HRFI and LRFI groups. Volcano dots of DEGs. The red dots indicate upregulated DEGs, the orange dots indicate downregulated DEGs, and the blue dots shows genes not significantly altered.

The gene expression levels for the eight sequencing libraries are listed in Supplementary Table S2. Genes were detected in all samples (FPKM >1), and 45 DEGs were identified; the expressions of 229 and 216 genes were upregulated and downregulated, respectively, in the HRFI group (Figure 1).

### 3.4 GO and KEGG analysis

**FIGURE 2:**
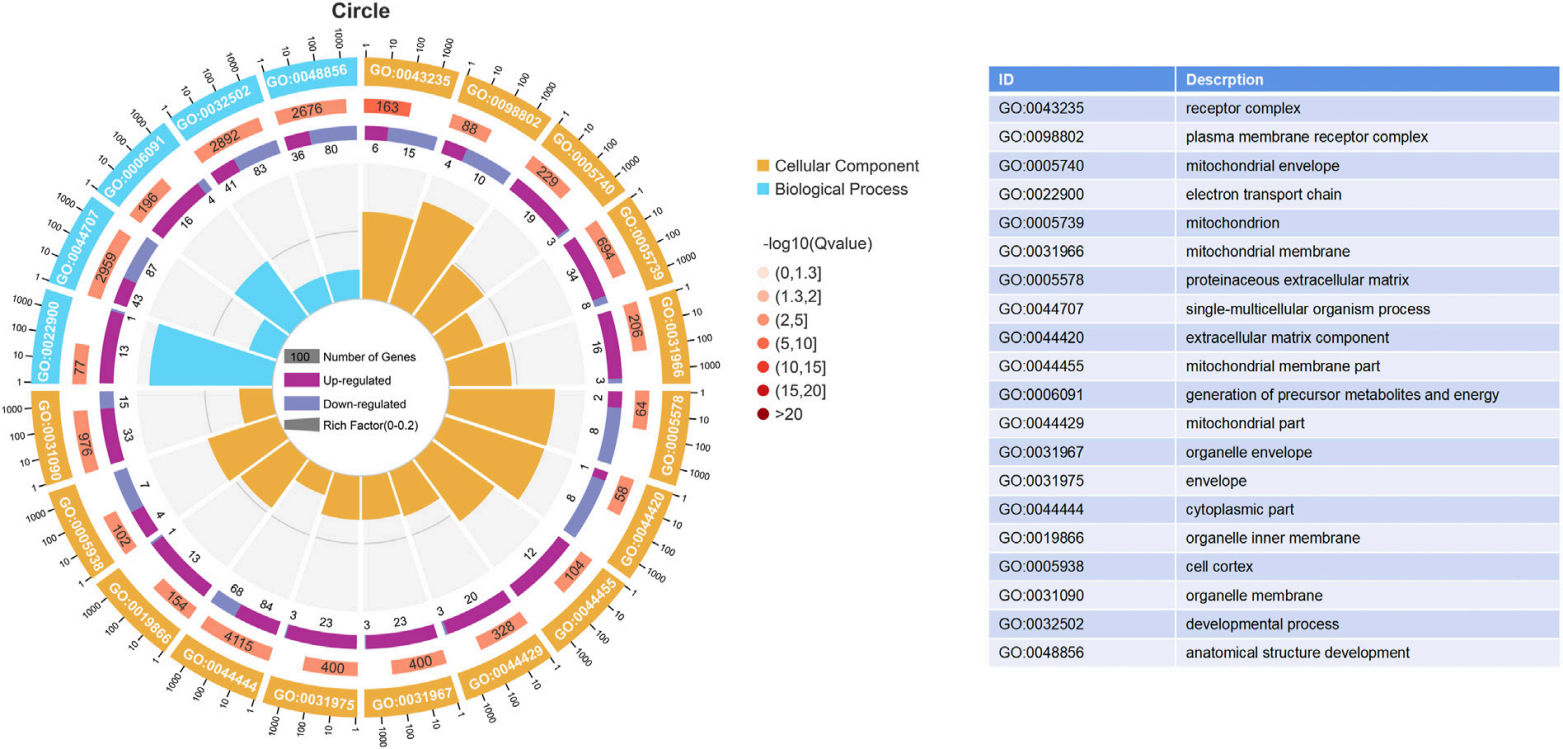
Enrich gene ontology (GO) terms based on DEGs between the HRFI and LRFI groups. The first lap indicates the top 20 terms, and the number of the genes corresponds to the outer lap. The second lap indicates the number of genes in the genome background and *p*-value for enrichment of the differentially expressed genes (DEGs) for the specified GO terms. The third lap indicates the DEG number. The fourth lap indicates the rich factor of each GO term.

**FIGURE 3:**
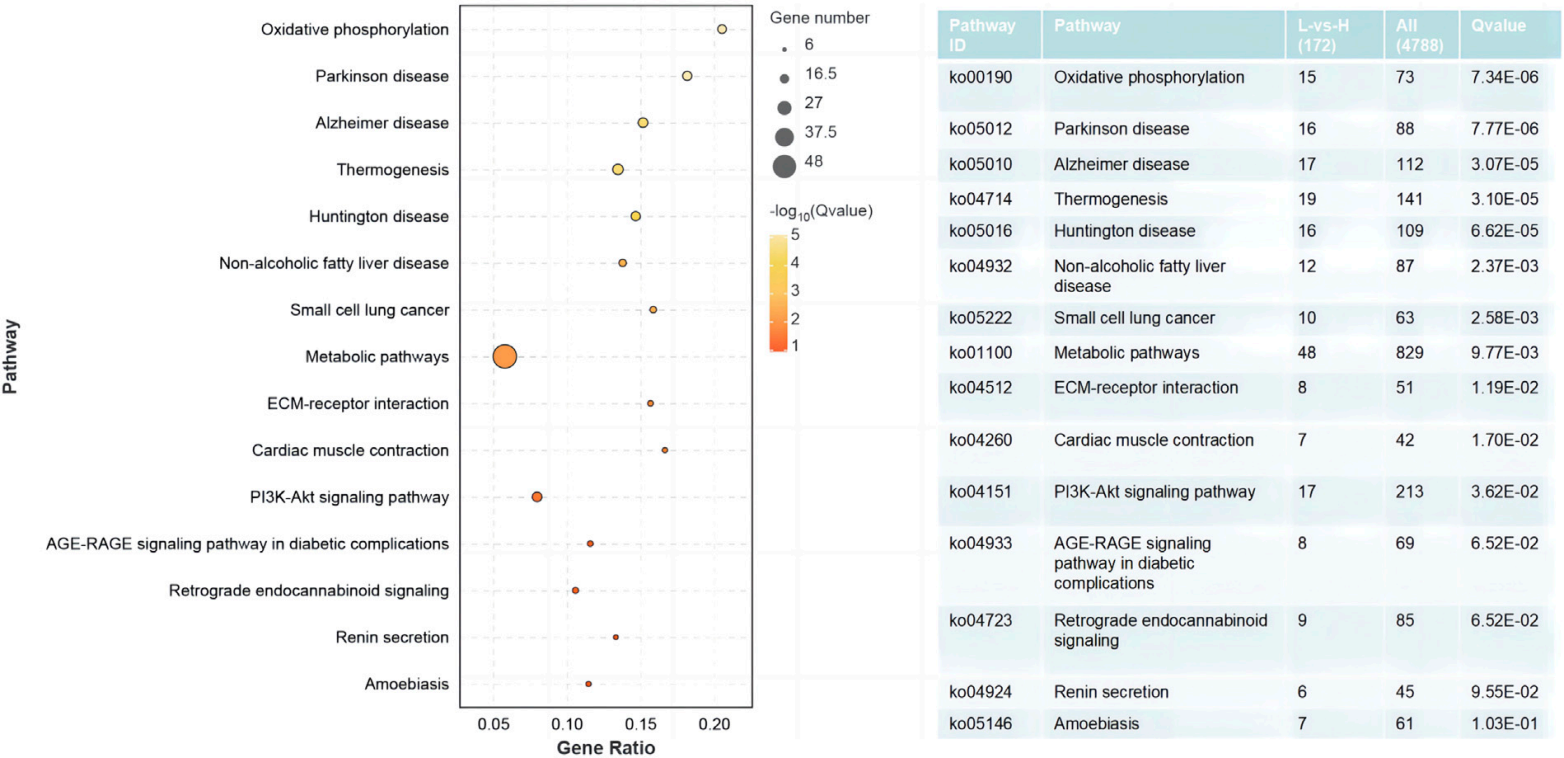
Significantly enriched KEGG pathways from DEGs between the HRFI and LRFI groups.

The results of the GO enrichment and KEGG pathway analyses are displayed in Supplementary Tables S4, S5. Forty-nine GO terms related to biological processes were significantly enriched, including metabolic, oxidation–reduction, ribonucleotide, ribose phosphate, and nucleoside triphosphate metabolic processes. Furthermore, number of GO terms related to molecular function and cellular components (such as the mitochondrial envelope, mitochondrion, and oxidoreductase activity) were significantly enriched (Figure 2). KEGG pathway analysis identified 11 significantly enriched pathways, including oxidative phosphorylation and PI3K-Akt signaling pathways, and ECM-receptor interactions that are closely related to energy metabolism (Figure 3).

### 3.5 GSEA

**FIGURE 4:**
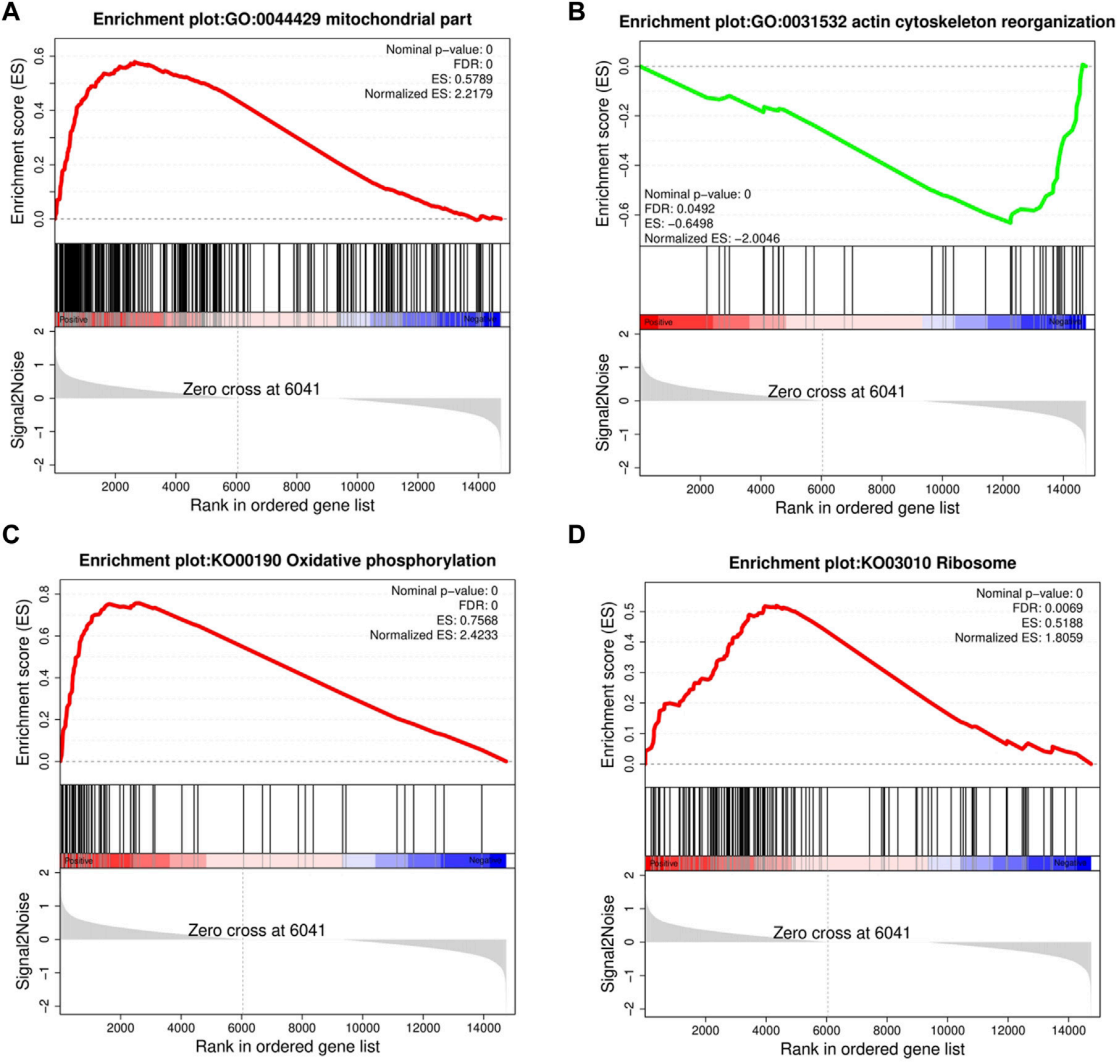
Gene set enrichment analysis (GSEA). GSEA was performed in the HRFI and LRFI groups. The GSEA algorithm calculates an enrichment score reflecting the degree of overrepresentation at the top or bottom of the ranked list of the genes included in the gene set in a ranked list of all genes present in the RNA-seq dataset. A positive enrichment score (ES) indicates gene set enrichment at the top of the ranked list; a negative ES indicates gene set enrichment at the bottom of the ranked list. The analysis demonstrates that **(A)** mitochondrial part, **(C)** Oxidative phosphorylation, **(D)** Ribosome are enrich in HRFI groups, while **(B)** actin cytoskeleton reorganization are enrichment in LRFI groups.

GSEA provided insufficient evidence to determine the molecular mechanisms of feed efficiency. Therefore, GSEA was performed to study the functions of all genes using gene sets from GO and KEGG-based lists (Supplementary Table S6). Overall, 445 GO and 39 KEGG-based gene sets were significantly enriched (|NES|>1, FDR<0.05). Positive and negative NES values represent higher expression levels in the HRFI and LRFI groups, respectively. The GO-based list showed that the higher expression gene sets in the HRFI group were primarily related to mitochondria and adenosine triphosphate (ATP) synthesis, whereas those in LRFI group were involved in ECM structural constituents, actin cytoskeleton reorganization, and growth factor binding. The KEGG-based list showed that the high expression gene sets in the HRFI group were primarily related to oxidative phosphorylation and the tricarboxylic acid (TCA) cycle, and those in the LRFI group were related to carbohydrate metabolism, signal transduction, and immune response (Figure 4; Table 3).

**TABLE 3:** Gene set enrichment analysis (GSEA) between HRFI and LRFI.

Gene set		NES	FDR	Higher expression in HRFI or LRFI
GO-based list (C5, CC, C5.BP, C5.MP) (Top 20)				
GO:0044455	mitochondrial membra`ne part	2.4360373	<0.001	HRFI
GO:0022900	electron transport chain	2.4230578	<0.001	HRFI
GO:0098798	mitochondrial protein complex	2.387794	<0.001	HRFI
GO:0098800	inner mitochondrial membrane protein complex	2.3862855	<0.001	HRFI
GO:0070469	respiratory chain	2.3428142	<0.001	HRFI
GO:0019236	response to pheromone	2.3026083	<0.001	HRFI
GO:0005743	mitochondrial inner membrane	2.296939	<0.001	HRFI
GO:0005740	mitochondrial envelope	2.2783267	<0.001	HRFI
GO:0031966	mitochondrial membrane	2.2694876	<0.001	HRFI
GO:0019866	organelle inner membrane	2.2621088	<0.001	HRFI
GO:0005746	mitochondrial respiratory chain	2.2349386	<0.001	HRFI
GO:0044429	mitochondrial part	2.2178912	<0.001	HRFI
GO:1990204	oxidoreductase complex	2.1585555	9.21E-05	HRFI
GO:0098803	respiratory chain complex	2.1450255	1.71E-04	HRFI
GO:0016503	pheromone receptor activity	2.109333	2.00E-04	HRFI
GO:0005747	mitochondrial respiratory chain complex I	2.1084182	1.87E-04	HRFI
GO:0022904	respiratory electron transport chain	2.106282	1.76E-04	HRFI
GO:0030964	NADH dehydrogenase complex	2.0895336	2.33E-04	HRFI
GO:0009055	electron carrier activity	2.0749488	2.52E-04	HRFI
GO:0005201	extracellular matrix structural constituent	−2.22948	<0.001	LRFI
KEGG-based list (C2.CP:KEGG)				
KO00190	Oxidative phosphorylation	2.423335	<0.001	HRFI
KO05012	Parkinson disease	2.3917017	<0.001	HRFI
KO04714	Thermogenesis	2.1306872	<0.001	HRFI
KO05016	Huntington disease	2.0541394	<0.001	HRFI
KO05010	Alzheimer disease	1.9485307	4.36E-04	HRFI
KO04932	Non-alcoholic fatty liver disease	1.8222557	0.006488033	HRFI
KO03010	Ribosome	1.8059274	0.006924233	HRFI
KO00020	Citrate cycle (TCA cycle)	1.7520487	0.015041439	HRFI
KO00250	Alanine, aspartate and glutamate metabolism	1.7237763	0.019311652	HRFI
KO00640	Propanoate metabolism	1.6465011	0.04506531	HRFI

Note: NES, normalized enrichment score; FDR, false discovery rate.

A positive NES indicates gene set enrichment in the HRFI group; a negative NES, indicates gene set enrichment in the LRFI group.

### 3.6 PPI networks of DEGs

**FIGURE 5:**
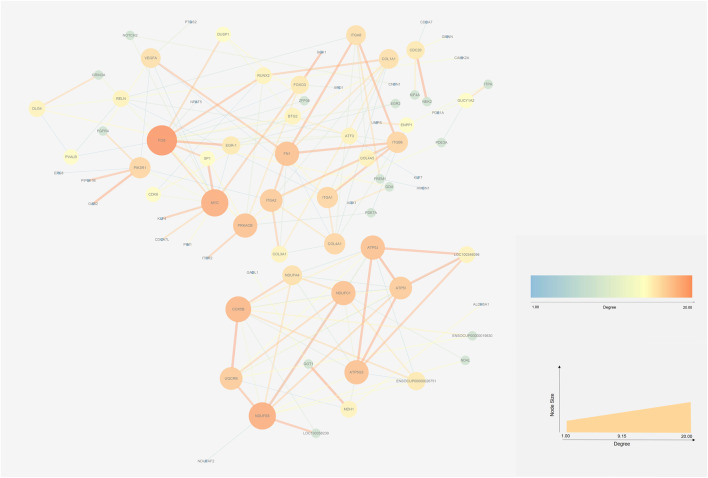
Protein-protein interaction (PPI) networks of DEGs between the HRFI and LRFI groups. The node represents the DEGs. Node size indicates the level of degree of each gene.

**FIGURE 6:**
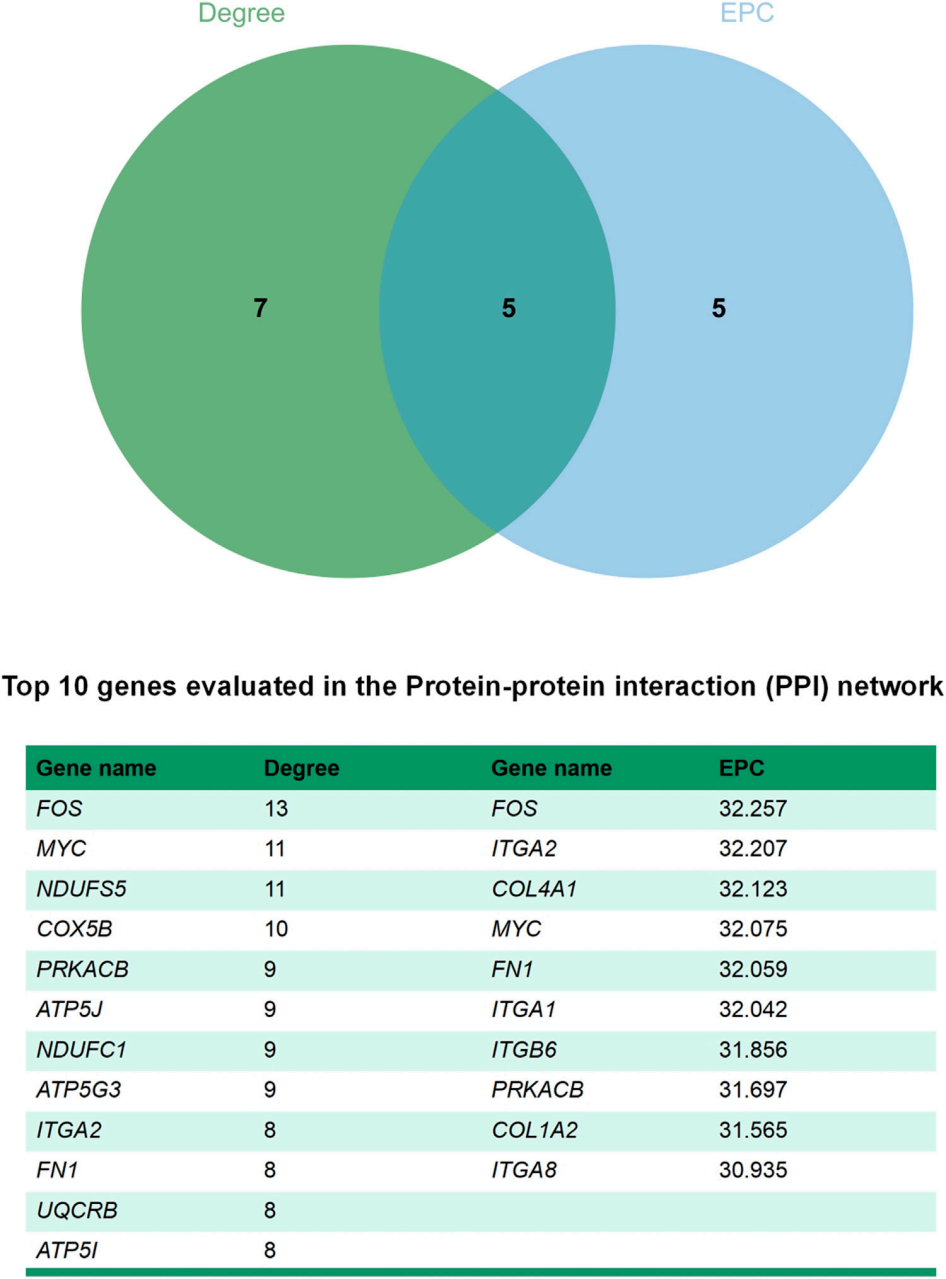
Venn plot identify significant hub genes generated by two centrality methods. Two methods—Degree and EPC were applied to identify significant hub genes. Different colors denote divergent algorithrns. The intersections indicate the common DEGs. The elements common to all methods were identified as the 5 core genes: *FOS*, *MYC*, *PRKACB*, *ITGA2*, and *FN1*.

The STRING database (https://string-db.org/) and Cytoscape v3.7.2 (Shannon et al., 2003)were used to integrate a potential network of DEGs in rabbit skeletal muscle that may lead to RFI differences. The PPI network comprised 127 nodes and 183 edges (Figure 5). The CytoHubba plugin was used to identify the top hub genes and the top 10 DEGs evaluated in the PPI were identified using two centrality methods (Degree and EPC). The intersections of these two algorithms were combined and a Venn plot was generated to identify hub genes (jvenn (inra.fr)) (Figure 6). The five hub genes that exhibited the highest degree of biological regulation between LRFI and HRFI rabbits were *FOS*, *MYC*, *PRKACB*, *ITGA2*, and *FN1*.

### 3.7 Validation of RNA-seq results

**FIGURE 7:**
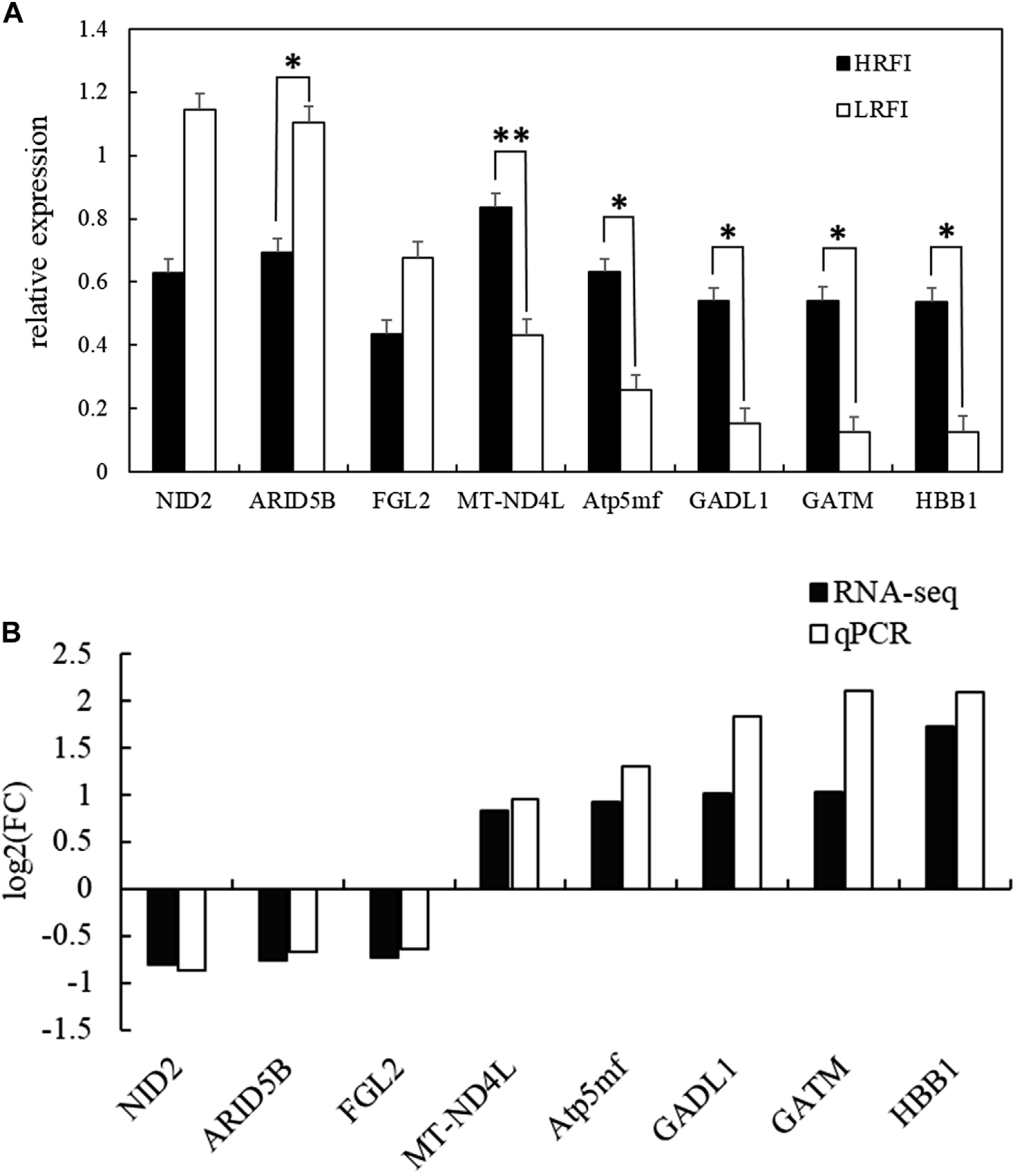
Validation of the RNA-seq results of eight DEGs via quantitative RT-PCR. **(A)** Verification of eight randomly selected DEGs via qRT-PCR (data are presented as means ± SEM; * means *p*-value <0.05, ** means *p*-value <0.01). **(B)** Comparison between the qRT-PCR results and sequencing results.

Eight DEGs, *NID2*, *ARID5B*, *FGL2*, *MT-ND4L*, *ATP5MF*, *GADL1*, *GATM*, and *HBB1*, were selected at random to validate the RNA-seq expression profiles via RT-qPCR using RNA samples from the HRFI (*n* = 4) and LRFI (*n* = 4) groups. The expressions of *NID2*, *ARID5B*, and *FGL2* were upregulated in the LRFI group, whereas those of *MT-ND4L*, *ATP5MF*, *GADL1*, *GATM,* and *HBB1* were upregulated in the HRFI group (Figure 7). The RT-qPCR expression patterns of these genes were consistent with those observed via RNA-seq.

## 4 Discussion

Our analyses of two groups of rabbits with excessive HRFI and LRFI revealed that rabbits in the LRFI group consumed less feed at the same growth rate, were more feed efficient, and had a lower FCR than the rabbits in the HRFI group. However, there were no differences in initial and final BW, ADG, or MBW between the two groups, consistent with the findings of previous studies on cattle (Nkrumah et al., 2004), lambs (Zhang et al., 2023), chickens (Metzler-Zebeli et al., 2017; Yang et al., 2020a) and ducks (Bai et al., 2022). Our results indicated that RFI selection in rabbits increased feed efficiency by reducing feed consumption without affecting rabbit growth performance. RFI is independent of BW and ADG, therefore can be used as an accurate, sensitive index to assess feed efficiency and improve animal genetic programs by eliminating the effects of different growth stages.

We identified 445 DEGs in the *M. longissimus thoracis et lumborum* of the two groups (229 with upregulated expressions and 216 with downregulated expressions) from the sequencing data. Several biological terms related to mitochondrial parts, energy metabolism, and immune function were revealed following GO annotation of the DEGs. Arguably, most genes associated with these terms are key influencers of feed efficiency in rabbits. Further, KEGG pathway analyses indicated that oxidative phosphorylation, the PI3K-Akt signaling pathway, and the ECM-receptor interaction signaling pathway were critical for mediating body metabolism. ECM-receptor interactions primarily regulate intracellular signal transduction and mediate interactions with cell adhesion receptors to modulate epithelial cell adhesion, motility, and growth (Levental et al., 2009). The ECM is a crucial component of tissue architecture and plays a key role in adipogenesis and meat quality (Taye et al., 2018; San et al., 2021; Shao et al., 2022). The PI3K-Akt signaling pathway is activated by various cellular stimuli or toxic insults and may regulate fundamental cellular functions, including transcription, translation, proliferation, growth, and survival (Peltier et al., 2007) and is involved in RFI variation in cattle (Yang et al., 2021) and shrimp (Dai et al., 2017). This study identified *COL1A, COL2A, FN1,* and *RELN* genes in the focal adhesion parts, which considered to be part of ECM components that mediate certain mechanisms involved in the PI3K-Akt signaling pathway.

The GSEA results of the GO list showed that all genes with high expression in the HRFI group were predominantly related to mitochondria and ATP synthesis. Thus, changes in the expression of genes associated with mitochondrial function are potential main drivers of rabbit feed efficiency. Over 95% of the cellular energy is produced by mitochondria via the TCA cycle and oxidative phosphorylation (Tzameli, 2012). Similarly, oxidative phosphorylation and the TCA cycle were significantly enriched in gene sets with high expression in the HRFI group based on the KEGG-based list. Thus, we propose that HRFI rabbits need more energy than LRFI rabbits to maintain normal life activities, leading to ATP synthesis, and that mitochondria number was potentially higher in the HRFI group than that in the LRFI group. LRFI rabbits may be more efficient than HRFI rabbits owing to the downregulation of mitochondrial function. These results are consistent with those obtained in previous transcriptome sequencing of skeletal muscle tissues from high- and low-RFI pigs (Vigors et al., 2019). Meanwhile, the high expression gene sets in the LRFI group were mostly involved in actin cytoskeleton reorganization, cell proliferation, and differentiation based on the GO-based list. Furthermore, carbohydrate metabolism, signal transduction, and immune response were the most significantly enriched gene sets according to the KEGG-based list, indicating that LRFI rabbits may be more efficient than HRFI rabbits in terms of energy utilization during muscle growth, which is consistent with the findings of previous studies (Horodyska et al., 2019; Yang et al., 2020b; Hou et al., 2020). Recent literature demonstrates that immune response is strongly associated with feed efficiency. For example, pigs with high feed efficiency have been shown to induce a more effective hepatic response to inflammatory stimuli than pigs with low feed efficiency (Horodyska et al., 2019). Cows selected for feed efficiency may have improved stress-coping abilities and immune responsiveness (Aleri et al., 2017). Similarly, LRFI pigs have an increased energy-saving mechanism in the intestinal innate immune response to immune challenges (Vigors et al., 2016). Therefore, our findings demonstrate that LRFI rabbits may be more robust and may better respond to infection than HRFI rabbits, consistent with the reports of previous studies.

The PPI networks constructed with DEGs in the current study identified the RFI candidate markers. The top centrality hub genes were *FOS*, *MYC*, *PRKACB*, *ITGA2*, and *FN1*. *FOS* is a proto-oncogene in mammals that forms the heterodimer complex Activator Protein-1 (AP-1) with c-Jun (Hou et al., 2019). *FOS* plays a vital role in the regulation of cell growth, division, proliferation, and differentiation and programmed cell death (Milde-Langosch, 2005). FOS/AP-1 is one of the earliest known transcriptional effectors in adult muscle stem cells. *FOS* accelerates the transition of stem cells from quiescence to activation via an early activated FOS/ART1/mono-ADP-ribosylation pathway, that is, essential for stem cell regenerative responses (Almada et al., 2021). Further, *FOS* gene phenotypic variation may be considered a marker of skeletal muscle fiber and metabolic traits in pigs (Reiner et al., 2002). *MYC* transcriptionally regulates many cellular processes and pathways, including cell growth, proliferation, and differentiation (Adhikary and Eilers, 2005). In mouse skeletal muscle, *MYC* overexpression stimulates skeletal muscle ribosome biogenesis and protein synthesis. In contrast, decreased *MYC* expression in mice reduces BW and growth rates (Mori et al., 2021). In the present study, we found that *FOS* and *MYC* were highly expressed in the LRFI group, thus confirming that LRFI rabbits may have more active proliferation and differentiation of skeletal muscle cells. *PRKACB* is a key effector of cAMP/PKA-induced signal transduction, which is involved in numerous cellular processes such as cell proliferation, apoptosis, metabolism, and differentiation (Chen et al., 2013). In addition, *PRKACB* serves as a potential biomarker of adipocyte lipolysis (Ji et al., 2020). *ITGA2* is an oncogene that may be important in cell migration, invasion, survival, and angiogenesis (Lian et al., 2018). In T cells, *ITGA2* may affect T-cell growth and proinflammatory cytokine expression (He et al., 2021) and it is overexpression may induce the activation of the PI3K/Akt signaling pathway (Liu et al., 2022). Similarly, *FN1* is known for its pivotal role in activating the PI3K/Akt signaling pathway. Fibronectin 1 (encoded by *FN1*) is a macromolecular glycoprotein that plays a vital role in cell adhesion, migration, proliferation, and differentiation (Ma et al., 2023). The *FN1* gene is involved in signaling pathways associated with immune processes (Li et al., 2022).

This study had some limitations. First, a larger sample size should be used for RNA-seq. Second, additional functional experiments should be conducted for verification of this study’s findings.

In conclusion, comparative analysis of the transcriptomes of *M. longissimus thoracis et lumborum* from HRFI and LRFI rabbits revealed *FOS*, *MYC*, *PRKACB*, *ITGA2*, and *FN1* as potential candidate genes that affect feed efficiency in rabbits. Several biological GO terms related to mitochondrial function, energy metabolism, and immune function were significantly enriched. Moreover, oxidative phosphorylation and the PI3K-Akt and ECM-receptor interaction signaling pathways were identified as the key signaling pathways for feed efficiency in rabbits. Additionally, the expressions of genes and pathways related to mitochondrial function were upregulated in HRFI rabbits, whereas those of genes and pathways related to immune response and energy metabolism were upregulated in LRFI rabbits. Our results explain the differences in RFI between the two RFI groups and will help improve feed efficiency in Wannan Yellow rabbits to ultimately enhance meat production.

## Data Availability

The datasets presented in this study can be found in online repositories. The names of the repository/repositories and accession number(s) can be found below: https://www.ncbi.nlm.nih.gov/, PRJNA978018.
